# Whole-Genome Duplication and Genome Instability in Cancer Cells: Double the Trouble

**DOI:** 10.3390/ijms24043733

**Published:** 2023-02-13

**Authors:** Tsz Yin Lau, Randy Y.C. Poon

**Affiliations:** 1 Division of Life Science, Hong Kong University of Science and Technology, Clear Water Bay, Hong Kong, China; 2 State Key Laboratory of Molecular Neuroscience, Hong Kong University of Science and Technology, Clear Water Bay, Hong Kong, China

**Keywords:** centrosome, genome instability, kinesin, mitosis, ploidy, tetraploidization

## Abstract

Whole-genome duplication (WGD) is one of the most common genomic abnormalities in cancers. WGD can provide a source of redundant genes to buffer the deleterious effect of somatic alterations and facilitate clonal evolution in cancer cells. The extra DNA and centrosome burden after WGD is associated with an elevation of genome instability. Causes of genome instability are multifaceted and occur throughout the cell cycle. Among these are DNA damage caused by the abortive mitosis that initially triggers tetraploidization, replication stress and DNA damage associated with an enlarged genome, and chromosomal instability during the subsequent mitosis in the presence of extra centrosomes and altered spindle morphology. Here, we chronicle the events after WGD, from tetraploidization instigated by abortive mitosis including mitotic slippage and cytokinesis failure to the replication of the tetraploid genome, and finally, to the mitosis in the presence of supernumerary centrosomes. A recurring theme is the ability of some cancer cells to overcome the obstacles in place for preventing WGD. The underlying mechanisms range from the attenuation of the p53-dependent G_1_ checkpoint to enabling pseudobipolar spindle formation via the clustering of supernumerary centrosomes. These survival tactics and the resulting genome instability confer a subset of polyploid cancer cells proliferative advantage over their diploid counterparts and the development of therapeutic resistance.

## 1. Introduction: Whole-Genome Duplication in Normal and Cancer Cells

The propagation of diploid genomes requires meticulous control of genome duplication and chromosome segregation. Limiting genome duplication to once per cell cycle is critical for maintaining genome stability in human cells. Errors in this process can trigger various types of genome damage and instability. A dramatic example of genome instability involves whole-genome duplication (WGD) in which the entire genome is doubled to create a tetraploid. WGD is one of the most common genomic abnormalities in cancers [1]. The genome is intrinsically unstable after WGD, both due to DNA damage during interphase as well as the frequent unequal segregation of chromosomes during mitosis.

Paradoxically, polyploids can be found in a subset of normal cell types including hepatocytes, megakaryocytes, myoblasts, and trophoblasts [2]. Mechanisms including cell fusion, endomitosis, endoreplication, and cytokinesis failure can generate polyploids through uncoupling of the checkpoint pathways that halt cell cycle progression or induce apoptosis after WGD [3]. Many cancer cells, however, develop mechanisms that can overcome these checkpoints. Moreover, they can often execute relatively faithful mitosis in the presence of extra centrosomes.

Here, we will chronicle the events following WGD in human cells. Starting from abortive mitosis, tetraploids can proceed to the replication of the tetraploid genome and eventually into mitosis in the presence of supernumerary centrosomes. We will detail the current understanding of the genome instability associated with WGD that may allow cancer cell evolution and increase in fitness. Strategies such as centrosome clustering that enable some cancer cells to maintain a relatively stable genome after WGD will also be discussed. Finally, how WGD can be exploited in cancer therapies will be highlighted.

## 2. Mitotic Failures Leading to WGD

**Figure 1:**
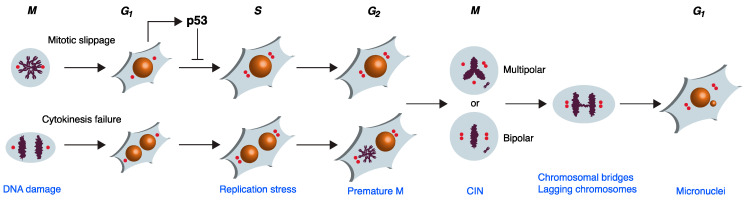
Genome instability following mitotic failure and WGD. Multiple opportunities are present throughout the cell cycle after mitotic failure for triggering genome instability. Cell cycle phases are indicated at the top and the associated genome instability is indicated at the bottom. Cells can exit mitosis without proper division through mitotic slippage or cytokinesis failure. In either case, the tetraploid G_1_ cells receive double the number of chromosomes and centrosomes compared to diploid G_1_ cells. A p53-dependent G_1_ checkpoint is induced due to DNA damage triggered by the abortive mitosis or through extra centrosome-mediated activation of PIDDosome or Hippo pathways. In the absence of the p53-dependent checkpoint, the cells can duplicate the DNA and centrosomes (red dots). Nonetheless, DNA damage is produced during S phase due to replication stress. In tetraploids derived from cytokinesis failure, DNA damage can also be induced in the G_2_ nucleus, which is forced into premature mitosis due to exposure to the mitotic environment of the neighboring nucleus within the same cell. Finally, the presence of supernumerary centrosomes can result in multipolar spindles during the following mitosis. Alternatively, pseudobipolar spindles can be formed by the clustering or inactivation (not shown in the Figure) of centrosomes. The high rate of syntelic and merotelic attachments in both multipolar and pseudobipolar mitosis promotes chromatin bridges and lagging chromosomes during anaphase. Chromatin bridges and lagging chromosomes contribute to genome instability by causing CIN and the formation of micronuclei, which act as yet another source of DNA damage and genome instability (other possible outcomes of multipolar and pseudobipolar mitosis are not shown). See text for details.

Several abnormal mitotic processes including mitotic slippage or cytokinesis failure can trigger WGD (Figure 1). Underlying these mitotic defects are various chromosome segregation problems. For example, unattached or incorrectly attached kinetochores to the spindles can delay mitotic exit and eventually induce mitotic slippage. Likewise, chromosome segregation errors can promote cleavage furrow regression and facilitate cytokinesis failure. Here, we will focus on tetraploidization induced after abortive mitosis in human cells. Readers are referred to recent reviews for more information on other tetraploidization mechanisms in cancer cells such as endoreduplication or virus-mediated cell–cell fusion [4,5].

Mitotic entry is driven by an engine composed of cyclin-dependent kinase 1 (CDK1) and its activating subunit cyclin B1 [6]. At the end of mitosis, cyclin B1 is destroyed by the ubiquitin ligase anaphase-promoting complex/cyclosome (APC/C) loaded with the targeting subunit CDC20 [7]. Activated cyclin B1–CDK1 mediates its own destruction by stimulating the activity of APC/C^CDC20^. The degradation of other proteins by APC/C also facilitates mitotic exit and progression into the next cell cycle. For example, the degradation of securin releases separase, which in turn cleaves cohesin complexes to facilitate sister chromatid separation [8]. APC/C-dependent proteolysis of geminin releases CDT1, enabling CDT1 to form the prereplicative complex for licensing the next round of DNA replication [9].

During normal mitosis, the dynamic nature of microtubules facilitates the efficient capturing of kinetochores by the spindles. Activation of APC/C^CDC20^ is initiated only when all the chromosomes have achieved accurate bipolar attachment to the spindles. Unattached kinetochores or the absence of tension between the paired kinetochores activates a spindle-assembly checkpoint (SAC), which inhibits APC/C^CDC20^ through the conversion of MAD2 from an open conformation (O-MAD2) to a closed conformation (C-MAD2). This helps to maintain an active cyclin B1–CDK1 environment during mitosis [10]. Once all the kinetochores are properly attached, new C-MAD2 is no longer generated from the kinetochores. The existing C-MAD2 is converted back to O-MAD2 by a process involving p31^comet^ and TRIP13 [11]. These mechanisms release APC/C^CDC20^ from inhibition by the SAC, allowing the cell to exit mitosis.

Agents that disrupt microtubule dynamics can lead to protracted activation of the SAC and mitotic arrest [12]. Classic examples include spindle poisons that attenuate microtubule depolymerization or polymerization (e.g., taxanes and vinca alkaloid, respectively). The outcome after prolonged mitotic arrest varies greatly between different cancer cell lines as well as between individual cells from the same cell line [13]. Apoptosis can be induced during mitotic arrest, possibly due to the progressive accumulation of apoptotic activators and/or the loss of apoptotic inhibitors, although the precise identities of these mitotic death signals remain elusive [14]. On the other hand, cells can exit mitosis precociously without proper chromosome segregation and cytokinesis in a process termed mitotic slippage. The central event of mitotic slippage appears to be a gradual degradation of cyclin B1 [15], correlated with a weakening of the SAC over the course of mitotic arrest [16]. Competition between the rate of cyclin B1 degradation (which affects the rate of mitotic slippage) and the accumulation of death signals (which affects the rate of mitotic cell death) is likely to determine the fate of a cell during prolonged mitotic arrest [13]. Cell death occurring both during mitotic arrest and following mitotic slippage is termed mitotic catastrophe [17]. An important implication is that if additional checkpoints are not present (see below), cells can evade mitotic catastrophe by undergoing WGD. Subsequent genome duplication and mitosis pave the way to further genome instability.

Cytokinesis failure is another major route towards WGD. Physical hindrance including chromosome nondisjunction [18] and chromatin bridges [19] can delay cytokinesis and promote cleavage furrow regression. Aurora B appears to be part of the sensor that responds to unsegregated chromatin at the cleavage site [19]. It has been estimated that these chromosomal segregation defects occur at a remarkably high frequency of ~1% in dividing somatic cells and at an even higher incidence in transformed cells [20,21]. A major culprit of chromatin bridges and lagging chromosomes is merotelic chromosome attachment (when a kinetochore is attached to microtubules emanated from two spindle poles). As merotelic attachment can satisfy the SAC, anaphase can be carried out even with incorrect spindle attachment [22].

Another causal factor of cytokinesis failure is the presence of damaged DNA during mitosis. The G_2_ DNA damage checkpoint normally prevents mitotic entry after DNA damage [23]. Deficiency of the checkpoint enables mitosis to occur in the presence of damaged DNA, and as a result, often generates chromosome fragments or entire lagging chromosomes that obstruct cytokinesis [24]. In fact, cells receiving persistent DNA damage can bypass mitosis entirely and undergo WGD [25]. Furthermore, the incorrect fusion of chromosomes during the repair of double-stand breaks (DSBs) can lead to the formation of chromatin bridges [26].

## 3. Overcoming G_1_ Checkpoint Arrest after Mitotic Failure

After mitotic slippage or cytokinesis failure, the first hurdle that tetraploids need to overcome is to pass through the G_1_ restriction point to initiate DNA replication [27]. It was initially proposed that cells are prevented from entering S phase following mitotic slippage or cytokinesis failure by a p53-dependent tetraploidy checkpoint [28]. Nevertheless, the nature of this checkpoint has since been disputed [29,30,31]. Instead of detecting the increase in ploidy per se, it is likely that the p53-dependent G_1_ arrest and/or senescence after tetraploidization is due to DNA damage inflicted during the previous aberrant mitosis [32]. Hence, the checkpoint may, at least in part, be a DNA damage checkpoint instead of one that detects extra chromosomes. Furthermore, p53 can also be activated after tetraploidization by extra centrosomes independent of DNA damage (see below).

More recent attempts to standardize various types of cell death defined mitotic catastrophe as cell death or irreversible cell cycle arrest associated with aberrant mitotic activity [33]. As G_1_ arrest after tetraploidization is generally a response to the previous dysregulated mitosis, it can also be classified as mitotic catastrophe.

The classic G_1_ DNA damage checkpoint involves the inhibition of CDK2 by the p53-p21*^CIP1/WAF1^* pathway. In essence, DNA damage activates sensors that facilitate the activation of the PI-3 (phosphoinositide 3-kinase)-related protein kinases ATM and ATR. ATM/ATR then activates CHK1 or CHK2 and together activate and stabilize p53 by relieving MDM2-mediated inhibition, culminating in the accumulation of the p53 transcriptional target p21*^CIP1/WAF1^* and the inhibition of G_1_ cyclin–CDK2 complexes. As phosphorylation of pRb by several G_1_ cyclin–CDK pairs (cyclin D–CDK4/6, cyclin E–CDK2, and cyclin A–CDK2) is required to release the transcription factor E2F for G_1_-S transition, the inhibition of cyclin–CDK2 complexes by p21*^CIP1/WAF1^* leads to G_1_ arrest [34]. Transcriptome profiling revealed signatures of p53 pathway activation at the early stages of tetraploidization [35]. Accordingly, G_1_ arrest after tetraploidization has been shown to be overcome by overexpression of cyclin D2 [35] or CDK2 [36]. As another crucial tumor-suppressing function of p53 is in the activation of apoptosis, the suppression of WGD by the p53 pathway is also likely to rely on apoptosis [37].

The p53 pathway can also be activated by excessive centrosomes independent of DNA damage. Extra centrosomes activate p53 by stimulating the activity of the PIDDosome multiprotein complex, leading to caspase-2-mediated cleavage of MDM2 [38]. Extra centrosomes in tetraploids also activate the Hippo pathway. After cytokinesis failure, extra centrosomes alter small G protein signaling (including RHOA and RAC1) to promote the activation of LATS2 kinase [39]. In addition to inhibiting the transcriptional regulators YAP and TAZ in the canonical Hippo pathway, active LATS2 then binds and inhibits MDM2, thereby indirectly leading to the stabilization of p53 [40].

In agreement with the crucial role of p53 in preventing the proliferation of tetraploids, the depletion of p53 in nontransformed RPE1 cells allows tetraploids to enter S phase after cytokinesis failure [39]. Downregulation of p53 can rescue tetraploid development in mice [41]. The absence of p53 also acts synergistically with tetraploidization to promote aneuploidy and tumorigenesis in mouse mammary epithelial cells [31].

Not all WGD cancers have a compromised p53 pathway. Despite the fact that p53 mutations and WGD are two of the most common molecular abnormalities in cancer, nearly half of the WGD cancers contain wild-type p53 [1]. Among these, more than 30% of the wild-type p53 WGD-positive cancers do harbor defects in the pRb-E2F pathway including pRb mutations and cyclin E amplification [1]. In other words, more than 30% of WGD cancers in fact have an intact G_1_ DNA damage checkpoint. Why some cancer cells can circumvent the p53-dependent checkpoint after WGD is a major unanswered question.

Mounting evidence indicates that whether the G_1_ DNA damage checkpoint is activated after tetraploidization is, at least in some cases, inextricably linked to the way that tetraploids are generated. For example, mitotic slippage is typically induced experimentally by exposing cells to microtubule inhibitors. During prolonged mitotic arrest, DNA damage is induced at telomeres in these cells due to telomere deprotection [42]. By contrast, inducing cytokinesis failure experimentally does not necessarily involve prolonged mitotic arrest and therefore is not coupled with DNA damage in the following G_1_ [29,30,31].

## 4. Genome Instability during the First Interphase after Mitotic Failure

Studies with yeast [43,44] and mammalian cells [31,45] have provided compelling evidence that WGD increases genome instability. A seminal study by Fujiwara et al. indicates that tetraploidization of p53-null mouse mammary epithelial cells induces aneuploidy and tumorigenesis [31]. As described above, the presence of p53 normally suppresses S phase entry in tetraploids. During the early stages of tetraploidization, the lack of p53 promotes the survival of chromosomally unstable subtetraploids, leading to genome instability and transformation [46].

It has become increasingly clear that WGD-mediated genome instability is induced as early as in the interphase immediately following tetraploidization. The mitotic defects that result in mitotic failure and tetraploidization are sources of DNA damage. For example, cells undergoing protracted mitotic arrest such as after exposure to microtubule inhibitors develop DNA damage at telomeres due to telomere deprotection from the loss of the shelterin complex [42]. The depletion of telomeres can produce dicentric chromosomes, which develop into chromatin bridges connecting the daughter cells and finally acquire DSBs through nuclear envelope rupture and exposure to cytosolic nucleases during interphase [47].

DNA trapped in the cytokinesis furrow might break and thereby trigger a DNA damage response in the daughter cells [48]. In normal human cells, the abscission checkpoint is responsible for delaying the final abscission of the intercellular canal that connects the two daughter cells until chromatin bridges are resolved [49]. The checkpoint involves Aurora B activity at the midzone and stabilization of the cytoplasmic canal [50]. Checkpoint-deficient cells or cells with unstable intercellular canals can result in chromosome breakage by the abscission machinery [50]. Cleavage furrow regression can also occur in the presence of lagging chromosomes or after unsuccessful resolution of chromatin bridges, producing tetraploid G_1_ cells [19,51].

A variety of mitotic perturbations including cytokinesis failure can precipitate replication stress during the subsequent S phase, giving rise to DSBs in the daughter cells [52]. High rates of DNA damage that occur during S phase after tetraploidization are caused by perturbation of DNA replication dynamics [53,54]. This is attributed to a shortage of proteins necessary for DNA replication, contrary to what is expected from the doubling of RNA and protein contents after WGD [53]. Consistent with this notion, overexpression of the transcription factor E2F1 (which positively regulates many DNA replication factors) can alleviate the levels of DNA damage in tetraploids [53]. This is also consistent with the upregulation of gene expression of the DNA repair pathways after WGD in mouse fibroblasts [55] and tetraploid primary cancers [53].

## 5. Mechanisms for Mitigating Supernumerary Centrosomes after WGD

Centrosomes are formed by a pair of centrioles surrounded by amorphous pericentriolar material (PCM). Each daughter cell receives one centrosome after mitosis. After the physical splitting of paired centrioles (centriole disengagement) during telophase/early G_1_, the centrosome linker is then established during G_1_ (including recruitment of C-Nap1 and rootletin). During S and G_2_, centrioles are duplicated and progressively recruit critical PCM components. At late G_2_, the two fully mature centrosomes separate through centrosome disjunction (dissolving of centrosome linker) and, finally, centrosome separation [56].

Centrosome duplication is highly choreographed during the cell cycle to ensure centrosomes are duplicated only once per cell cycle. Major upstream regulators of centrosome duplication include the polo-like kinase PLK4 [57]. The G_1_-specific cyclin E–CDK2 complexes are at least in part responsible for the coordinated initiation of centrosome and DNA duplication [58,59]. Accordingly, the inhibition of CDK2 activity by the p53-dependent checkpoint (see above) prevents both DNA replication and centrosome duplication after tetraploidization [60]. A direct role of p53 in the regulation of centrosome duplication has also been proposed [61].

Supernumerary centrosomes are generally poorly tolerated in human cells due to the resulting multipolar mitosis. Nevertheless, a subset of tetraploids possess mechanisms to either silence or coalesce excess centrosomes, enabling them to undergo bipolar mitosis [62]. In addition to tetraploidization, supernumerary centrosomes can be acquired by a process of centrosome amplification, when centrosomes duplicate more than once per cell cycle [63]. It should be noted that our limited knowledge on the determinants of centrosome inactivation and clustering is obtained from studies using both WGD and centrosome amplification models. A potential caveat is that the mechanisms of mitigating supernumerary centrosomes in the two conditions may not be identical.

In their seminal work, Basto et al. reported that supernumerary centrosomes can be partially inactivated in *Drosophila* neuroblasts [64]. The silenced centrosomes exhibit reduced PCM assembly and capacity to form microtubule aster. Similarly, extra centrosomes are inactivated in Sak (*Drosophila* homologue of PLK4)-overexpressing disc cells. Co-overexpression of moesin (a member of the ERM complex involved in regulating cortical contractility) restores the recruitment of centrosomal proteins to the silenced centrosomes and leads to abnormal spindle formation [65]. How cell cortex organization is linked to centrosome inactivation remains to be established. While some events including phosphorylation [66] and interaction of tubulin with the centrosomal protein CPAP [67] are known to regulate the recruitment of PCM proteins to centrosomes, the process is likely to be regulated by multiple factors progressively during the cell cycle.

Compared to centrosome inactivation, more studies have been focused on potential regulators of centrosome clustering after WGD. Nonetheless, it should be emphasized that, as clustered interphase centrosomes tend to contain altered microtubule nucleation, it is possible that centrosome clustering is in part regulated by centrosome inactivation initiated during interphase [67,68]. Pioneering work by Quintyne et al. revealed that supernumerary centrosomes can be clustered to form pseudobipolar spindles [69]. Subsequently, several genome-wide RNAi screens in *Drosophila* and mammalian models were performed in search of regulators of the process. These analyses used either cells that contained extra centrosomes (*Drosophila* S2 [70,71] and oral squamous cell carcinoma UPCI-SCC-114 [72]) or colon adenocarcinoma DLD1 cells that had undergone cytokinesis failure [73]. These putative regulators of centrosome clustering are summarized in Table 1. A striking feature of these results is the diversity of proteins being implicated, underscoring the intricate linkages between centrosome clustering to many processes.

A major class of suppressors of multipolar spindle formation is microtubule motor proteins. For example, the minus-end-directed mitotic kinesin KIFC1 (HSET) has long been known to mediate centrosome clustering in cells harboring excessive centrosomes [74,75]. This is countered by outward forces between centrosomes generated from the antagonistic plus-end-directed mitotic kinesins KIF11 (kinesin-5; Eg5) and KIF15. In this connection, APC/C is believed to be a regulator of centrosome clustering through KIF11 degradation [73]. The minus-end-directed motor dynein–dynactin further provides inward forces that pull extra centrosomes together [69]. The effects of some kinesins on centrosome clustering may be more indirect. For example, KIF23 is involved in the formation of cleavage furrow during cytokinesis. It is possible that KIF23 (and other potential regulators of centrosome clustering that are involved in cytokinesis such as Anillin, ECT2, and PRC1; Table 1) promotes multipolar mitosis due to the consequence of cytokinesis failure of the previous mitosis.

Finally, it is possible that centrosome clustering can be influenced by the physical environment after tetraploidization. There is evidence that the polyploid chromosomes themselves can act as a physical barrier to reduce centrosome clustering [76].

**Table 1:** Regulators of clustering of supernumerary centrosomes.

Processes	Proteins	Centrosome Clustering	RNAi Screens	References	Processes	Proteins	Centrosome Clustering	RNAi Screens	References
**Actin-related**	ARP3	**+**	K			CENP-T	**+**	L	
	Cofilin	**−**		[77]		INCENP	**+**	G, L	
	LIMK2	**+**	D			MAD2	**+**	K	[64]
	MYHK-IIb	**+**	D			MPS1	**+**		
	MYLK	**+**	D			NDC80	**+**	L	
	MYO9B	**+**	D			RCC1	**+**	K	
	MYO10	**+**		[78]		SKA1	**+**	D	
	MYO15	**+**	K			SKA2	**+**	D	
	RhoA	**+**	K			SKA3	**+**	D	
	TESK1	**+**	D			SPC24	**+**	L	
	WASL	**+**	K			SPC25	**+**	L	
**Augmin**	HAUS1	**+**	L			SPINDLY	**+**	D	
	HAUS3	**+**	L			Survivin	**+**	L	
	HAUS6	**+**	L	[75,79]	**Microtubule-related**	ASPM	**+**	K	
**Centrosome**	AURKA	**+**		[80]		CKAP5/ch-TOG	**+**	K, L	[81]
	Calmodulin-1	**+**	K, D			CLIP-170	**+**	K	
	Calmodulin-3	**+**	K, D			FES	**+**	D	
	CEP110	**+**		[82]		HURP	**+**		[83]
	CEP164	**+**	L			TACC	**+**	K	[81]
	CEP215	**+**		[74]		MAST1	**+**	D	
	CPAP	**+**		[67]		MAST4	**+**	D	
	HSP70	**+**		[84]		TPX2	**+**	L	
	NEK6	**+**		[84]		Tubulin	**+**	K, L	
	NuMA	**−**		[69]	**Miscellaneous**	CERT1	**+**	D	
	PLK4	**+**	D			KCC2G	**+**	D	
**Cell adhesion**	CDH11	**+**	L			IFT52	**+**		[85]
	DDR2	**+**	D			IFT88	**+**		[85]
	E-cadherin	**−**		[86]		LGN	**+**		[78]
	Keratin-5	**−**		[87]		MLKL	**+**	D	
	MPP2	**+**	D			PRPS1	**+**	D	
	MPP3	**+**	D			TSSK6	**+**	D	
	PEAK1	**+**	D		**Signaling**	CAMK2α	**+**	D	
	Periostin	**+**	L			CAMK2δ	**+**	K	
	Plakoglobin	**−**		[87]		CDK1/2	**+**		[88]
	Tenascin-X	**+**	L			ILK	**+**		[81]
	VE-cadherin	**+**	L			LATS1	**+**	D	
**Chromatid cohesion**	SCC4	**+**	D			MAP3K7	**+**	D	
	SGO1	**+**	L			MAP3K11	**+**	D	
	Sororin	**+**	L			MAP4K5	**+**	L	
**Cytokinesis**	Anillin	**+**	L			MARK3	**+**	K, D	
	ECT2	**+**	L			MASTL	**+**	D	
	PRC1	**+**	L			MERTK	**+**	D	
**DNA damage response**	ATM	**+**	D	[89]		p53	**+**		[90]
	ATR	**+**		[89]		PIK3Cβ	**+**	K	
	NEK4	**+**	D			PIP4K2β	**+**	D	
	PARP1	**+**				PIP4K2γ	**+**	D	
	PARP6	**+**		[91]		PP2A-Aα	**−**		[92]
**Dynein-dynactin**	ARP1	**+**	D			STAT3	**+**		[93]
	DYNC1H1	**+**	D, L			STK10	**+**	D	
	DYNC1I2	**+**	D			STK33	**+**	D	
	Dynein	**+**		[69]		TAOK1	**+**	D	
	DYNLRB1	**+**	D			TAOK2	**+**	D	
	LIS1	**+**	D			TRPM7	**+**	D	
	p22	**+**	D			TSSK2	**+**	D	
	p150^Glued^	**+**	D			TSSK3	**+**	D	
**Kinesin**	KIF2C/ MCAK	+/− *	K	[76]	**Ubiquitination**	APC/C	**+**		[73]
	KIF10/ CENP-E	**+**	K			APC3/ CDC27	**+**	D	
	KIF11	**−**		[73,94]		APC5	**+**	D	
	KIF15	**−**		[94]		APC6/ CDC16	**+**	D	
	KIF18A	**+**		[95]		APC8/ CDC23	**+**	D	
	KIF20A	**+**		[96]		APC10	**+**	D	
	KIF23/ MKLP1	**+**	G, L			APC11	**+**	D	
	KIF24	**−**		[97]		FBX4	**+**	L	
	KIFC1/ HSET	**+**	G, K	[64,74,75]		Polyubiquitin-C	**+**	L	
**Kinetochore**	AURKB	**+**	G, L			USP8	**+**	K	
	Borealin	**+**	D, G, L			USP28	**-**		[98]
	BUB1	**+**	K			USP31	**+**	K	
	CEPN-A	**+**	K			USP54	**+**	L	

D: Drosopoulos et al. (2014) [73]; G: Goshima et al. (2007) [70]; K: Kwon et al. (2008) [71]; L: Leber et al. (2010) [72]. Potential regulators of supernumerary centrosome clustering identified from RNAi screens and other studies are shown (* discrepancy in the results from different studies).

## 6. WGD Induces Genome Instability during Mitosis

WGD after mitotic failure produces daughter cells containing tetraploid DNA contents and two centrosomes. In the absence of checkpoints including the loss of p53 function (see above), both the DNA and centrosomes can be further duplicated during the subsequent S phase [99]. Pseudobipolar spindles can be formed in the presence of supernumerary centrosomes by centrosome inactivation or clustering (see above). Without these mechanisms, cells advance into mitosis with multipolar spindles and segregate their genetic materials unevenly into daughter cells [3].

Tetraploids exhibit higher chromosomal instability (CIN) than diploids even when they are able to form bipolar spindles. In yeast, the mismatches in the scaling of the size of the spindle pole body, spindles, and kinetochores in tetraploids result in a high rate of syntelic and monopolar kinetochore attachments to the spindle pole [44]. Furthermore, merotelic kinetochore-microtubule attachments are increased in mammalian tetraploid cells that can form pseudobipolar spindles through the clustering of supernumerary centrosomes compared to diploids [100,101]. The presence of extra centrosomes is sufficient to promote merotelic attachments before centrosome clustering can take place [101]. A principal outcome of unresolved merotelic attachment is the presence of chromatin bridges and lagging chromosomes during anaphase [22,101]. These further contribute to genome instability by causing aneuploidy upon missegregation as well as by forming micronuclei [102,103]. Micronuclei appear to be poorly equipped in maintaining stable DNA, resulting in extensive DNA damage and chromosome fragmentation within them. Integration of micronuclei into the genome during the next mitosis may further increase genome instability by inducing massive rearrangements such as chromothripsis [102].

Finally, uncoordinated entry into mitosis may also play a role in genome instability after WGD. DNA damage can be induced by unscheduled entry into mitosis, such as after premature activation of the mitotic cyclin B1–CDK1 complexes [17]. In tetraploids derived from cytokinesis failure, the two nuclei can enter mitosis out of synchrony. In this scenario, DNA damage is induced in the nucleus that is forced into mitosis due to exposure to the mitotic environment of the neighboring nucleus within the same cell [104].

## 7. Size Matters: Promotion of Tumorigenesis after WGD

Polyploidization generated from WGD is believed to serve important functions in normal tissue development in addition to being a key event in tumorigenesis [105]. Unlike in organisms such as fish and amphibians, polyploidization is poorly tolerated in humans. Nonetheless, polyploidy occurs physiologically in specialized cell types including hepatocytes, megakaryocytes, myoblasts, and trophoblasts [2]. The general perception is that the larger cell volume rendered by the increased ploidy is beneficial in a range of physiological functions including wound healing and tissue regeneration [106]. Examples include the production of platelets from large polyploid megakaryocytes formed by endomitosis [107]. The increase in myofiber size and contractile strength after cell–cell fusion of myoblasts is another example [108].

Another advantage of WGD involves the increase in and potential redundancy of genetic materials. WGD has been speculated to be an essential step during evolution. Ohno proposed in his seminal work that WGD provides the primary source of redundant genes for new evolutionary opportunities [109]. Within a shorter time scale, normal cells in an organism may use a similar strategy to expand gene functions. For example, ploidy reversal in polyploid hepatocytes is believed to be a mechanism for generating genetic diversity, enabling hepatocytes to adapt to xenobiotic or nutritional injury [110]. In a similar manner, cancer is increasingly being recognized as a rapidly evolving system. Nearly 30% of cancer patients had tumors that underwent WGD [1,111,112]. The extra sets of chromosomes after WGD may supply cancer cells with a depot of genetic materials to buffer deleterious somatic alterations and facilitate clonal evolution. In agreement with this, WGD appears to be enriched in cancer with a high deleterious alteration rate, including in lung squamous cell carcinoma and triple-negative breast cancers [113]. Transcriptional reprogramming after WGD may also confer polyploid cancer cells proliferative advantage over their diploid counterparts and the development of therapeutic resistance [111,112].

The majority of cancer cells are highly aneuploid, displaying dynamic karyotypic changes including the gain or loss of whole chromosomes. WGD can be found in the early stages of many cancers, including Barrett’s esophagus [114,115] and cervical carcinoma [116]. More recent genome sequencing analysis indicated that many cancers display evidence of WGD. The frequency of WGD varies markedly by cancer type, from more than 50% in germ cell tumors and small cell lung cancer to about 5% in non-Hodgkin lymphomas and gastrointestinal neuroendocrine tumors [1]. The prevailing view is that tetraploids are transient intermediates that can promote CIN [117]. Moreover, evidence from the transformation of epithelial cells from mouse salivary glands indicates the appearance of tetraploid cells before they undergo a period of CIN [45]. Evidence from in vitro and animal models also suggests a link between WGD induced by viral-mediated cell fusion and cancer [118]. CIN accelerates the acquisition of oncogenes and deletion of tumor suppressor genes and is sufficient to initiate tumorigenesis in mammalian cells [119].

Following WGD, additional rounds of mitotic failure and WGD are expected to destabilize the genome further. The ability of cells to execute cytokinesis properly reduces as ploidy increases, giving rise to multinucleated giant cells [120]. Although whether giant cells still retain proliferative potential is still controversial, there is evidence that these cells can undergo multipolar mitosis to return to near-diploid states and acquire stemness [121]. These studies suggest that the multistep process of escaping cell death through polyploidization followed by depolyploidization may account for tumorigenesis and tumor relapse after initial efficient cancer therapy [122].

Collectively, the available evidence underscores the critical role of WGD in the initiation of CIN and neoplastic transformation. The increase in genome instability associated with WGD is likely to be a two-edged sword, promoting deleterious cell division in most cells but enabling cancer genome evolution to acquire growth advantages in a small subset of cells.

## 8. Large Targets: WGD and Cancer Therapies

WGD in cancer cells offers tantalizing opportunities for therapies that may enable selectivity against polyploid cancer cells while sparing normal diploid cells. At first glance, the increase in ploidy and cell volume in polyploid cells is expected to potentiate sensitivity to conventional chemotherapies. For example, higher DNA contents in polyploids render them more sensitive to ionizing radiation and topoisomerase inhibitors [123]. Furthermore, as polyploid cells are characterized by a relatively high basal level of DNA damage in comparison to diploid cells (see above), it is possible that the high intrinsic DNA damage can be exploited using conventional chemotherapies. Nonetheless, depolyploidization after WGD may rapidly reduce the differences in DNA contents and cell volume between normal and cancer cells [122].

Given the prevalence of centrosome amplification and clustering in cancer cells, it is not surprising that a major focus of therapeutic development has been on strategies that decluster extra centrosomes [62]. The idea is that detrimental multipolar mitosis can be triggered in cancer cells containing supernumerary centrosomes without affecting normal diploids. Although many regulators of centrosome clustering are potential drug targets (see Table 1), extensive work will be needed to translate these mechanistic insights into clinical benefits. One encouraging example is the mitotic kinesin KIFC1, whose inhibition preferentially sensitizes cancer cells through centrosome declustering [124,125,126].

Another approach is to look for genes that are essential in WGD cells but not in diploid cells. One example is KIF18A, a mitotic kinesin that regulates microtubule dynamics to suppress chromosomal oscillations at the metaphase plate. Dependency on KIF18A scales proportionally with the degree of aneuploidy, conferring its unique requirement in cells after extensive CIN [95]. KIF18A is an attractive therapeutic target because it appears to be dispensable in most normal cells, as suggested by evidence from *KIF18A*-knockout mice [127,128]. Microsatellite instability and mild alterations in chromosome number do not render cells prone to KIF18A loss, indicating that KIF18A might be particularly vital in maintaining genome stability after WGD [129,130]. Depletion of KIF18A increases the duration of mitosis, chromosome misalignment, lagging chromosomes in anaphase, and micronuclei formation in WGD cells [130]. The underlying mechanisms may involve the preferential enlargement of spindle size and increases in the magnitude of chromosomal oscillations after KIF18A depletion in WGD cells. These effects increase the propensity of chromosomes to lose their attachment to the mitotic spindles and to activate the SAC [130]. This is consistent with the findings that components of the SAC including BUBR1, MAD2, and BUB3 are among the most preferentially essential genes in WGD- or aneuploidy-positive cell lines [95,130]. Inhibition of the SAC using the MPS1 inhibitor AZ3146 induces mitotic delay, chromosome segregation errors, and micronuclei formation similar to KIF18A depletion [130]. Unlike KIF18A, however, most core components of the SAC are essential in normal cells. Notwithstanding, as the expression of SAC components is frequently altered in cancers, it is possible that the SAC is a useful target to explore for sensitizing WGD cancer cells. Moreover, as several SAC components such as MAD2 and BUB1 are implicated in the clustering of extra centrosomes (Table 1), targeting the SAC may have the additional benefit of promoting multipolar mitosis in WGD cells.

## 9. Concluding Remarks and Future Perspectives

A growing body of evidence reveals that WGD promotes genome instability at multiple levels. DNA damage and genome instability are already prevalent during the first cell cycle after tetraploidization. These range from the DNA damage generated during the initial mitotic failure to the replication stress during S phase, and finally, to the subsequent CIN mediated by multipolar or pseudobipolar mitosis (Figure 1). Thereafter, additional rounds of cell cycle and division are likely to exacerbate genome instability further.

One of the characteristic features of WGD is the multiple opportunities for the accumulation of DNA damage throughout the cell cycle. DNA damage can be triggered from the initial mitotic failure that leads to tetraploidization, ranging from telomeric deprotection during prolonged mitotic block to chromatin bridges that initiate cytokinesis failure. Replication stress associated with tetraploidization is another source of DNA damage. Finally, chromosome attachment defects associated with increased spindle size and extra centrosomes during the subsequent mitosis are coupled to DNA damage caused by chromatin bridges and lagging chromosomes. A major theme in genome instability after WGD is the attenuation of the p53 pathway in cancer cells, resulting in the overriding of the G_1_ checkpoint. Interestingly, the p53-independent G_2_ DNA damage checkpoint has not been reported to play a prominent role after WGD. The underlying mechanism will require further investigation. Activation of p53 by extra centrosomes through the PIDDosome or Hippo pathway may explain some of the cases. More work will also be required to understand the mechanistic basis of the relatively high percentage of WGD cancer cells that contained wild-type p53 (and functional pRb pathway). It is conceivable that WGD and extra centrosomes can be tolerated under some circumstances in vivo without triggering the activation of p53.

Many issues also remain to be resolved related to the clustering or inactivation of extra centrosomes after WGD. While many studies have provided compelling evidence of factors that can regulate centrosome clustering or inactivation, they were often performed with leveraging models containing amplified centrosomes rather than experimentally induced tetraploidization. Whether centrosome clustering is regulated by the same factors in diploids with centrosome amplification, induced tetraploids immediately after WGD, and stable tetraploids remains to be defined precisely. Further research is also required to validate the range of proteins suggested by the RNAi screens to provide a comprehensive picture of the regulators of centrosome clustering.

Given that WGD is inextricably linked to genome instability, a puzzling question is how tetraploids that arise physiologically can maintain a stable genome. One possibility is that the genome instability observed experimentally is predominantly due to the procedures that initiate WGD. Tetraploids produced under physiological conditions could be more stable than those generated in vitro. An alternative thought is that as most polyploids in vivo are engaged in terminally differentiated programs, maintaining genome stability in these cells may be less important than in dividing cells that produce progenies. Evidence supporting this notion includes the findings that the mitotic cycles of polyploids appearing during the regular course of development in *Drosophila* are inherently error-prone [131]. These are provocative ideas that will need to be investigated further.

Large gaps also remain to be filled in the area of therapeutic targeting of WGD cancer cells. In addition to exciting targets such as KIF18A, are there other proteins that are essential specifically in WGD cancer cells but not in normal diploid cells? Although the SAC is essential for normal cells, can it be exploited due to the differential expression of SAC components in WGD cancer cells? Nonetheless, the types of cancer that may benefit from this approach are likely to be those containing a high portion of relatively stable polyploids instead of those that contain unstable ploidy.
